# Tumor Cell Communications as Promising Supramolecular Targets for Cancer Chemotherapy: A Possible Strategy

**DOI:** 10.3390/ijms251910454

**Published:** 2024-09-27

**Authors:** Irina Alekseenko, Lyudmila Zhukova, Liya Kondratyeva, Anton Buzdin, Igor Chernov, Eugene Sverdlov

**Affiliations:** 1 Shemyakin-Ovchinnikov Institute of Bioorganic Chemistry of the Russian Academy of Sciences, 117997 Moscow, Russia; irina.alekseenko@gmail.com (I.A.); buzdin@oncobox.com (A.B.); igor.palich@gmail.com (I.C.); 2 National Research Center “Kurchatov Institute”, 123182 Moscow, Russia; 3 Department of Oncology, SBIH “Moscow Clinical Scientific and Practical Center Named After A.S. Loginov” DHM, 111123 Moscow, Russia; zhukova.lyudmila@rambler.ru; 4 World-Class Research Center “Digital Biodesign and Personalized Healthcare”, Sechenov First Moscow State Medical University, 119992 Moscow, Russia; 5 Oncobox LLC, 121205 Moscow, Russia

**Keywords:** cancer, tumor microenvironment, cell interaction, immunological synapse, crosslinking agents, next-generation chemotherapy, targeted therapeutics

## Abstract

Fifty-two years have passed since President Nixon launched the “War on Cancer”. Despite unparalleled efforts and funds allocated worldwide, the outlined goals were not achieved because cancer treatment approaches such as chemotherapy, radiation therapy, hormonal and targeted therapies have not fully met the expectations. Based on the recent literature, a new direction in cancer therapy can be proposed which targets connections between cancer cells and their microenvironment by chemical means. Cancer–stromal synapses such as immunological synapses between cancer and immune cells provide an attractive target for this approach. Such synapses form ligand–receptor clusters on the interface of the interacting cells. They share a common property of involving intercellular clusters of spatially proximate and cooperatively acting proteins. Synapses provide the space for the focused intercellular signaling molecules exchange. Thus, the disassembly of cancer–stromal synapses may potentially cause the collapse of various tumors. Additionally, the clustered arrangement of synapse components offers opportunities to enhance treatment safety and precision by using targeted crosslinking chemical agents which may inactivate cancer synapses even in reduced concentrations. Furthermore, attaching a cleavable cell-permeable toxic agent(s) to a crosslinker may further enhance the anti-cancer effect of such therapeutics. The highlighted approach promises to be universal, relatively simple and cost-efficient. We also hope that, unlike chemotherapeutic and immune drugs that interact with a single target, by using supramolecular large clusters that include many different components as a target, the emergence of a resistance characteristic of chemo- and immunotherapy is extremely unlikely.

## 1. Introduction—Chemotherapy: Fame and Failure

The year 2023 marks 52 years since 23 December 1971, when U.S. President Nixon declared what was later called the “War on Cancer” [1,2,3]. Large allocations were made. The President promised that, in five years, a cure for cancer would be available, and by the year 2000, the incidence of cancer would be halved [4]. This enthusiasm was based on successful experimental results in the treatment of children with acute leukemia. Since then, progress in this “war” is still being discussed, and the results on cancer mortality are presented in different ways—either as progress or as failure. As Howy Jacobs wrote, “Nixon’s war on cancer outlasted his presidency and his life. However, after forty years we still hope for the future progress of modern therapy, even though the number of publications on this topic during this period is so large that it will soon reach the moon” [5]. There is no doubt that the war against cancer, as defined by Nixon, was lost, and its combat targets were never defeated. All efforts to significantly reduce overall cancer mortality have essentially been not successful. A recent review stated: “Despite over a century of intensive efforts, the great gains promised by the War on Cancer nearly 50 years ago have not materialized” [6]. Donald Kennedy, who headed the US FDA and Stanford University, later described the war on cancer as “A Medical Vietnam” [3].

These conceptual failures have led to the fact that, despite the great merits of chemotherapy in the treatment of cancer and, especially, in the progress in our understanding of disease mechanisms, there has been a pronounced pessimism about its prospects as a medical strategy. Thus, in 2016, Agarwal ended his brief but extremely capacious review [7] (“Is cancer chemotherapy dying?”) with the words “Today, we are moving towards non-chemotherapeutic drug therapy of malignancy. Also, today we are moving from protocol-based treatment to personalized therapy based on prognostic markers, markers predictive of drug sensitivity/resistance, markers predictive of adverse events and molecular profiling. I have no hesitation in predicting that very soon, chemotherapy may play a very small role in the cure of cancer”. Also, in his very skeptical commentary, Bizzarri notes that chemotherapy has many defects, and its effectiveness causes skepticism [8]. And even such a recognized authority in the field of oncology as one of the authors of the “Cancer hallmarks” concept, Douglas Hanahan [2], in his Editorial “Rethinking the war on cancer” (which we will discuss in more detail later) notes: “The war on cancer has largely focused on mutant cancer cells, via chemotherapy and radiotherapy and, more recently, targeted therapy... Most of the time, however, cancers eventually find ways to circumvent such targeted strikes, adapting and then reemerging as expansive and often more aggressive growths. … The reality, however, is that targeted therapies are generally not curative or even enduringly effective, because of the adaptive and evasive resistance strategies developed by cancers under attack”.

However, there remain battlegrounds where chemotherapy maintains its dominance. For example, unlike in other cancers wherein the substitution of chemotherapy is possible, in germ cell tumors it is very difficult—the use of platinum drugs is necessary [9]. Also, at least in some cases, chemotherapies can augment tumor immunity, for example, by inducing immunogenic cell death [10,11,12], and this hints at the prospects of searching for optimal combinations of chemo and immunotherapy.

The use of the term “targeted” requires some explanation. Traditionally, cancer is treated by surgery, radiotherapy and chemotherapy. Chemotherapy originated from poisonous gases that were used during World War II. It has been successfully used to cure many otherwise fatal cancers, such as childhood acute lymphoblastic leukemia, Hodgkin’s lymphoma, testicular cancer and so on. Also, a significant increase in life expectancy in many other diseases such as high-grade non-Hodgkin’s lymphomas and multiple myeloma was encouraging. However, the problem with chemotherapy is that, in addition to destroying the cancer cell, it also causes widespread damage to other tissues, especially bone marrow, resulting in morbidity and even mortality. Therefore, enormous efforts of oncologists have been and continue to be aimed at increasing the specificity of the drug by directing its action to certain cancer-specific properties—targets. Regardless of whether targeting is carried out using chemical agents or genetic therapy, as we noticed as early as 2011 [13], the term “targeting” can be divided into two broad strategies: (1) the targeted destruction of tumors as a whole exploiting the features shared by all cancers, for example, relatively fast mitotic cell division, and (2) using the ideology of molecular targeted therapy, targeted at certain molecular component(s) or pathways presumably crucial for maintenance of a certain cancer type [13,14]. Previously, we analyzed in detail the reasons why molecular-targeted medicine is objectively doomed to failure [13,15]. The above quote [2] provides even more reasons why this failure is inevitable. There, Hanahan expresses an extremely important idea, which he then details in his article: “I suggest that much like in modern warfare, the war on cancer needs to have a battlespace vision”. In this paradigm it is “Space” not “target” that must be taken into account for effective therapy of such a multifaceted disease as cancer. We expressed the same idea in our recent review [15], pointing out the need for a multidimensional strategy.

And, nevertheless, as the authors of [16] quite rightly noted in their review, the efforts expended were not wasted: the “war” led to the accumulation of enormous (albeit still far from complete) information about the mechanisms of the occurrence and evolution of cancer. With a new understanding of the molecular mechanisms of disease progression, our knowledge of the disease is growing rapidly and giving rise to many new therapeutic approaches. In recent decades, various combinations of treatments [17] have been proposed that are currently being used to treat various types of cancer and were not common a few years ago (see, for example, [16]). At the same time, these authors, in our opinion, quite rightly point out that “chemotherapy remains a largely opted therapeutic option despite its known side effects on the patient’s physical and psychological health. Chemotherapeutic agents/pharmaceuticals served a great purpose over the past few decades and have remained the frontline choice for advanced-stage malignancies where surgery and/or radiation therapy cannot be prescribed due to specific reasons”.

Hanahan also indicates an extremely important issue, which is not always taken into account by researchers involved in fundamental aspects of cancer treatment: “Another sobering issue is the reality that many exciting new cancer treatments are very expensive... despite in many cases producing only transitory clinical benefit, posing serious cost–benefit dilemmas for patients, health insurers, and governments” [2]. We think that, in the case of cancer, the modern medical “sacred cow”, personalized medicine, due to the extreme heterogeneity of cancers, faces serious difficulties in its application and formulas; this is contrary to possible generalized principles of cancer medicine. Such approaches seem especially relevant against the backdrop of a rapidly growing population. In such conditions, big medicine should not avoid focusing on universal therapies. In the case of success of such a strategy, chemotherapy can be just an inexpensive and universal procedure, provided that a battlefield is found that ensures its supramolecular and universal action, i.e., applicable to different types of cancer.

In this review, we would like to present arguments for the hypothesis that not all possible potentials of cancer chemotherapy have been exhausted. Currently, the vast majority of procedures are aimed at *intracellular* processes, whether they involve the use of a higher rate of DNA replication or a cancer-specific increase or decrease in any specific components. As we discussed earlier, this route of targeted therapy has not yet resulted in a significant breakthrough in the modern cancer care that could be converted into a dramatic increase in life expectancy for cancer patients. One of the alternative, seemingly promising strategies may involve chemotherapeutic interventions aimed at disrupting the *intercellular* components of cancer, i.e., communication of cancer cells with their microenvironment. Unlike chemotherapeutic and immunotherapeutic drugs that act on a specific target, the emergence of critical resistance when acting on large supramolecular clusters composed of many different components is extremely unlikely.

## 2. Basic Definitions, Terms and a Brief Presentation of Available Information

### 2.1. Tumor Tissue Compartments

As a rule, tumor tissue is considered to consist of three parts: (1) the tumor core (TC, in other words, a tumor nest or tumor cluster), comprising the majority of tumor cells in a certain limited area; (2) the tumor stroma (TS) with abundant stromal components surrounding the TC; and (3) the invasive margin (IM), a ~1 mm transition zone between the TC and the TS. Cancer cells in the IM may have a partial epithelial–mesenchymal transition (EMT) phenotype, expressing some EMT-determining genes that are not expressed in the central core of tumors [18]. The IM plays a central role in orchestrating tumor–host interactions [19,20,21] (for a recent review, see [22]). The IM forms the front line of the tumor’s fight against the immune system, and the activity and density of immune cells in this zone may be higher than in the TC or the TS [19]. There is ambiguity in the use of the terms “stroma” and “microenvironment”. For simplicity, we will use the terminology used in Kalluri’s classic article [23]: “tumor stroma or tumor microenvironment. These are typically components of the host response to cancer cells, including the immune response. These terms are used interchangeably”.

### 2.2. Basement Membrane and Extracellular Matrix

The extracellular matrix (ECM) is an important component of the tumor microenvironment and is formed by a network of macromolecules in the extracellular space of tissues that provide a molecular scaffold for cell growth, survival, differentiation and migration. The ECM provides structural support to cells and tissues and plays a key role in the functional properties of cells. The ECM is divided into two groups: the basement membrane and the interstitial matrix. The basement membrane (BM) is formed by a thin layer of ECM at the interface between the epithelial or endothelial layer and connective tissue or surrounding nerves, adipocytes and muscle cells. The BM is primarily composed of laminin and collagen IV and serves as a structural barrier to cancer cell invasion, intravasation and extravasation. Cancer invasion through the BM is the initial stage of tumor dissemination and metastasis [24,25,26]. In epithelial cancers, cells must penetrate the BM to metastasize. When malignant epithelial cells have broken through the basement membrane and penetrated into the adjacent stroma to a depth of 1 mm or less, microinvasion is said to be present [27].

### 2.3. Tumor Microenvironment (TME)

The TME mainly includes the extracellular matrix of tumor immune cells (TICs), fibroblasts and secreted factors. TICs are often associated with sensitivity to immunotherapy and the prognosis of multiple cancers, but the prognostic role of individual cells in tumor prognosis is limited.

The mechanism of the breaching of human cancer cells into the BM remains little investigated. Until recently, it was believed that this required degradation by proteases, since the cells (~10 microns) were too large to penetrate the BM pores (several nanometers). An active role in this process belongs to the tumor epithelium, immune cells and cancer-associated fibroblasts (CAFs, see below) [28]. CAFs are thought to be capable of digesting BM components via matrix metalloproteinases (MMPs) [29,30]. In addition, recent studies also indicate a protease-independent BM invasion through physical forces generated by cancer cells, involving collective cellular interactions, proliferation, CAFs and myoepithelial and immune cells. In this hypothesis, disruption of the BM, like in metastasis, involves the cells pushing through the basement membrane into the stoma [26,31]. The ability of tumor cells to form various types of actin-rich protrusions, including invasive protrusions (invadopodia) and locomotor protrusions (lamellipodia [2D] or pseudopodia [3D]), may play an important role in BM disruption and tumor cell dissemination [26,32] (see below).

CAFs have also been reported to induce BM permeability through an MMP-independent mechanism; they remodeled the matrix by applying contractile forces, creating gaps between the BM fibers that allowed cancer cells to pass through [33]. However, all this information is preliminary and requires additional research [34]. It should also be noted that most tumors arise from adhesive epithelial cancer cells that have strong intercellular contacts. Metastasizing from the original tissue, epithelial tumor cells change their adhesive properties through the EMT process and synthesize proteins characteristic of mesenchymal cells, which leads to a decrease in the level of epithelial proteins and a decrease in the adhesiveness of intercellular contacts [35].

### 2.4. Paracrine and Juxtacrine: Two Types of Cell Signaling

The complexity of interaction networks of cancer and host cells is still far from being well understood [36,37]. Intercellular contacts which are crucial for the existence of the organism are also necessary for the emergence and evolution of tumors. Chemical and physical interactions between the cells mediate the exchange of information [38]. A fundamental analysis of the patterns of information exchange between cells was published in 2012 by Perrimon et al. [39] (see also more recent reports in [15,40,41]). Extracellular pathways are used first, for example, in the secretion of signaling entities (e.g., exosomes, cytokines and metabolites). Physical communication, in turn, describes direct interactions between cells [42].

Existing classifications of biochemical signaling processes define autocrine signals if the cell sends a signal to itself. Paracrine signaling occurs between cells situated near one another. Juxtacrine signaling takes place if cells are situated in close contact with each other. Finally, endocrine signaling represents the exchange of information, e.g., by hormones between distant cells or organs through the bloodstream [43].

In all cases, the ligands bind to the receptors of the cell receiving the signal. It is important to note that paracrine-signaling molecules are released into the extracellular space and then diffuse to reach the receptor of the receiving cell. By analogy with the gradient distribution of morphogens during development, in paracrine signaling, signal molecules secreted by the donor cell into the environment are distributed along a gradient with a concentration decreasing with distance from the donor. Accordingly, the probability of signal effectiveness decreases. Cells close to the ligand source receive its high concentration, whereas cells far from the ligand source receive low concentration. The physical properties of the ligand affect the final distribution of the ligand and hence the resulting signaling efficiency. Perrimon demonstrated this using paracrine signaling in Drosophila tissues. In the case of RTK, receptor–tyrosine kinase (RTK) ligands, and the ligands that activate the fibroblast growth factor (FGF) receptor, the signaling range is restricted to two to eight cell diameters. In contrast, the ligands for Wnt/Wg pathways can extend up to 30 cell diameters. We therefore propose that the preferred form of signaling from cancer cells to cells of the microenvironment is juxtacrine signaling, which requires only direct contact between cells and does not release signaling molecules into the extracellular space. Perrimon et al. distinguish three types of juxtacrine signaling: (a) a protein from one cell binds to its receptor on the surface of an adjacent cell, as occurs at immunological synapses (see below); (b) ECM glycoprotein and the membrane protein interact; for example, a receptor on one cell binds to its ligand on the ECM secreted by another cell; and (c) the signal is transmitted directly from the cytoplasm of one cell to the cytoplasm of an adjacent cell through a small tube [39,44]).

The latter case is characteristic of gap junctions, which, for the transport of ions and small soluble molecules, connect the cytoplasm of two neighboring cells through the clustering of tens to thousands of intercellular channels. Gap junctions are involved in a wide range of biological processes and consist of bonds that include six proteins—connexins [45]. Immunological synapse (IS), predominantly involving membrane ligands and receptors, is formed at the interface between a T cell and an antigen-presenting cell during the adaptive immune response, or a cancer cell, and a similar mechanism is thought to mediate the virological synapses [46].

Recently, an unexpected juxtacrine communication mechanism was identified that utilizes characteristic protrusions of plasma membrane cells to facilitate sensing of the extracellular environment, intercellular information exchange and materials processes for chemical signaling through direct contact over long distances [47,48]. Cells can use filopodia for this purpose—exploratory cytoplasmic projections consisting of F-actin [49]. Filopodia can extend over 800 µm and have a diameter of 100–500 nm [50].

Two types of intercellular connections can be formed: cytoneme and cell-connecting tunnel nanotubes (TNTs) [50]. The TNTs are F-actin-based structures that form direct cytoplasmic connections between distant cells. In immune cells, TNTs were shown to play an important role in signaling [51] by transporting DNA, mitochondrial DNA organelles, vehicles, exosomes, proteins, genetic material, ions and small molecules, viral RNA and non-coding RNA from cell to cell under physiological and pathological conditions [47,52,53]. TNTs have been observed in a variety of cell types, including neuronal, immune, cancer and stromal cells [54]. TNTs are long-range cytoplasmic intercellular channels for direct intercellular communication, independent of soluble factors. Uniquely, these structures enable the rapid exchange of cellular cargo between connected, non-contiguous cells, including organelles, vesicles, molecules, ions and pathogens [55]. The connections of multiple cells by TNTs form functional cellular networks. TNTs can also form a membrane junction between the donor cell and the target cell, which may promote immunogenic synapse formation [56] or gap junction signaling [57,58]. TNTs can be used by pathogens, especially viruses, to facilitate their spread.

**Figure 1:**
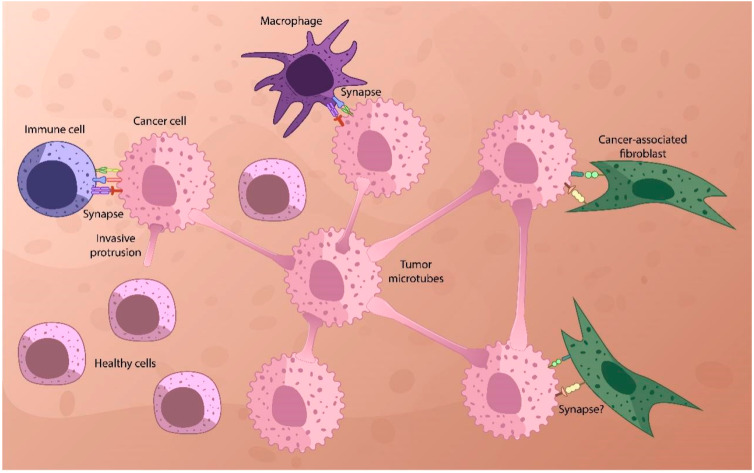
Tumor microtube network. In cancers, structures called *tumor microtubes* (TMs) and *tunneling nanotubes* (TNTs, see text) connect tumor cells and cells from microenvironments such as CAFs, allowing them to act as a single, organ-like unit. The network also involves immune cells forming synapses with cancer cells and, possibly, with CAFs [69]. The potential synapse between cancer cell and CAF is marked as “synapse?”.

TNTs are minimally present in adult healthy tissues (except maybe for the immune system) in physiological conditions (see Figure 1). It is suggested that TNTs are stimulated by different diseases and inflammatory stimuli and may be involved in disease transmission [59]. Most importantly, the existence of the TNTs between cancer cells and stromal cells ex vivo and in vivo has been reported [60]. Cancer cells form TNTs with other cancer cells as well as with microenvironmental cells [61,62,63]. This allows us to think about the therapeutic applications of this phenomenon. Cytonemes are specialized closed-ended actin-based signaling filopodia. Cytoneme signaling in cells occurs due to the interaction of a ligand produced by the signaling cell localized on a growing thin membrane protrusion, which is a cytonema, with the receptor of the cell that receives the signal.

In the case of a cytonema, there is no cytoplasmic connection between two cells; this type of connection is considered non-tubular. Cytonemes can reach lengths of several hundred microns (most mammalian cells have a diameter of 10 to 100 microns). To transmit signals, they use a specific ligand–receptor interaction between the tip of the cytoneme and the target cell. TNTs are different because they allow for the exchange of cell surface molecules and cytoplasmic contents between distant cells [49,55]. In solid tumors, cancer cells can be spread, which results in problematic communication through gap junctions or exosomes. Instead, TNTs and possibly other TME options can support direct physical contacts between the distant cancer cells.

The length of the filopodia is consistent with the radius of distribution of signal molecules secreted [39,64,65]. These protrusions can deliver signals in both ways: from the sender to the recipient and back [38,43,55,58,61,62,66,67]. The protrusions are heterogeneous [46] and can form, for example, TNTs or tumor microtubes (TMs), which consist of actin and function as intercellular bridges connecting a variety of cell types. TMs are longer and have a larger diameter compared with TNTs observed in vitro [49,55,57,65,68].

**Figure 2:**
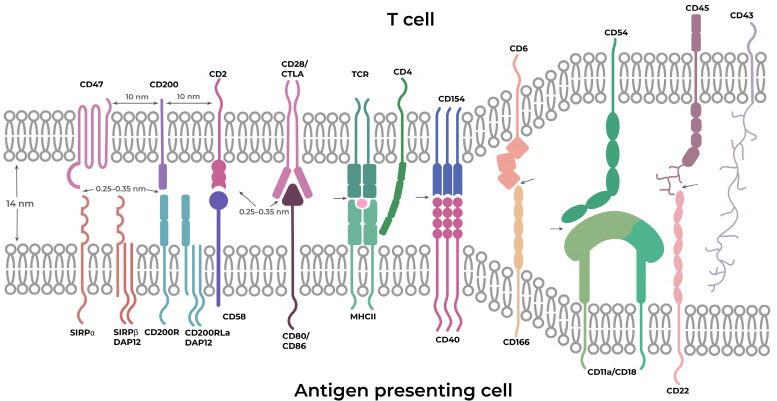
Schematic illustration of interactions between an antigen-presenting cell and a T cell. Approximate distances between neighboring synapse components on the same cell–cell interface surface were approximated according to the data from [78].

Synapses are specific intercellular interfaces that have a unique membrane organization and provide relevant signal transmission between contacting cells. Of all the methods of direct communication between the cells, synapses and the respective cell–cell interfaces appear to be among the most promising supramolecular targets for medical intervention [43,70]. This kind of contact, common to all types of immune cells, uses direct ligand–receptor adhesion for intercellular signaling [45,71,72,73,74,75]. Immunological synapse (IS) is a mechanism used for intercellular communication mediated by vesicular traffic, as well as a site for the active release of soluble molecules [76]. They are heterogeneous, starting with a variable width of cleft formed between T cells and antigen-presenting cells, whose average interfacial gap is ~14 nm (Figure 2), continuing with a variable length of the synapse itself from short (hundreds of nanometers) to very long interfaces, extending over several micrometers. Synaptic contacts have variable durations, starting from short transient contacts and continuing to quite long or even permanent neuronal contacts. The lateral organization of proteins within junctions plays an important role in the integration and regulation of signals from various sources. For example, during the formation of the IS, T-cell receptor (TCR) molecules quickly form submicron-sized microclusters, which work as sites of active signaling [70].

Another physical feature of cell–cell boundaries is a consequence of the different sizes of cell surface molecules and the limited size of the gap between membranes at the interfaces. The height of the proteins can determine whether they can be accommodated within an interface and participate in cell–cell interaction enhancement [70,77].

## 3. General Physicochemical Principles of Direct Intercellular Contacts

The extracellular protein–protein interactions observed between membrane-embedded receptor proteins vary from very strong to very weak. Intercellular recognition between two opposing membranes, therefore, requires large multivalent clusters consisting of hundreds or thousands of receptor molecules that increase the total avidity of the interaction with the ligands to a point high enough to prompt a signaling event [70,79]. The receptor–ligand signaling interactions are highly diverse. A common feature of the clusters is the close location of their components on the surface (about 10 nm). Another extremely important property of clusters is that their components interact cooperatively [15,70]. For example, T cells are capable of detecting a single ligand molecule on antigen-presenting cells; however, nearly ten such molecules or more will be necessary for T cell activation [80]. As for signaling by secreted ligands, the range of signaling elicited by the ligand-producing cell can span tens of cell diameters. Thus, the distribution of ligands over such a distance generates a graded signaling profile, which may lead to distinct responses depending on the distance from the source cell(s), rather than a simple on/off switch [15,39].

### 3.1. Extracellular Vesicles—Disputable Mean of Intercellular Communication

All cells release extracellular vesicles (EVs) that can be broadly classified into two categories, ectosomes and exosomes. Ectosomes pinch off the surface of the plasma membrane and embrace microvesicles, microparticles and large vesicles from ~50 nm to 1 μm in diameter. Exosomes are particles with a size from ~40 to 160 nm (average ~100 nm) in diameter [81]. According to a popular point of view, fibroblasts can be “educated” by cancer cells and in response secrete EVs and establish intercellular communication that possibly benefits cancer progression [82].

In this paradigm, EVs mediate intercellular communications by functioning as messengers because they contain as cargo various biomolecules, in particular, nucleic acids and proteins (for a recent review, see [82,83]). It has been hypothesized that the cargo being transferred from cell to cell may cause phenotypic changes in the recipient cells.

The hypothesis that EVs could transfer RNA-encoded information from cell to cell was put forward by Valadi et al. [84] in 2007. The title of this publication, “Exosome-mediated transfer of mRNAs and microRNAs is a novel mechanism of genetic exchange between cells”, speaks for itself. Actually, the authors demonstrated that EVs purified from murine cell lines do contain small RNAs, and these RNAs may also be found in human cells after exposure to these EVs. Due to limits in the sensibility and specificity of the experimental techniques used in their research, it was problematic to the authors to distinguish between the true and a false positive weak signals observed. Also, this experiment could be strongly impacted by the use of in vitro non-physiologically high concentrations of EVs not corresponding to the in vivo conditions. However, since then, quite a significant number of publications have described how EVs may participate in a molecular crosstalk among cancer and stromal cells. It should be added that this information was obtained mainly from the in vitro experiments, and adequate confirmatory in vivo studies have not yet been reported [85]. Note that there exist considerable difficulties in the EV purification and standardization of experimental protocols for determining the functionality of EVs for obtaining reliable evidence of intercellular trafficking [86].

In 2012, one of the authors of this review (E.S.) published a critical paper [87] questioning (1) whether data on RNA transfer obtained in vitro are physiologically relevant in terms of real-world in vivo molar concentrations, and, consequently, (2) whether the potential of EVs to deliver information (e.g., through nucleic acids) is physiologically relevant. The conclusion was that this is extremely unlikely. A sufficient number of objective analytical reviews appeared later, which in general agree with this point of view (for most recent reviews, see [85,86,88,89]). On the other hand, the flow of articles beginning with sentences like the following: “In cancer, EVs play an integral role in cell–cell communication and transfer pro-oncogenic molecules to recipient cells thereby conferring a cancerous phenotype” does not subside. However, experimental substantiation of such statements is clearly insufficient [85,86,88,89]). Still, the transportation of these particles through the mechanisms involving TNTs, the MT and synapses cannot be excluded [55,90].

During the evolution of a cancer, the transfer of information between cells occurs mainly through direct physical interactions between cells and through TNTs and MTs, although some contribution of paracrine information and information carried by extracellular vesicles cannot be excluded [48]. Recent findings in several organ systems show that cytoneme-mediated signaling transports signaling proteins along cellular extensions and targets cell-to-cell exchanges to synaptic contacts. This mechanism of paracrine signaling may be a general one that is used by many (or all) cell types in many (or all) organs. We briefly review these findings in this perspective. We also describe the properties of several signaling systems that have previously been interpreted to support a passive diffusion mechanism of signaling protein dispersion, but can now be understood in the context of the cytoneme mechanism [91].

### 3.2. Immune and Other Cells within Tumor Compartments: A Quick Glance

Tumor-infiltrating immune cells and stromal cells are thought to play a crucial role in tumor progression and response to therapy. Cytotoxic T lymphocytes (CTLs), myeloid antigen-presentation cells and CAFs have various preferable locations within tumors [19,92].

In colorectal cancer and metastatic pleural mesothelioma, there was a higher enrichment of immune infiltrate detected in the stroma compared with the other tumor cell compartment, but it was approximately equal in the tumor core and the IM [93,94]. The cancer ECM generally excludes some types of immune cells (for example, infiltrating CD8+ T cells which are the main effectors of anticancer immunity) but can accumulate the others, such as macrophages and neutrophils [95]. Recent results suggest that the content of tumor-infiltrating lymphocytes (TILs) may be essential for prognostic purposes [96]. For example, a positive prognostic role is played here by the CD8+ T cells that recognize and destroy tumor cells containing cancer neoantigens. In contrast, a negative role here belongs to Treg cells, M2 macrophages and myeloid-derived suppressor cells that can cause immunosuppression and at least thereby enhance tumor growth [97].

CAFs, one of the major components of the TME, originate from different predecessors and are functionally heterogeneous [98,99,100]. CAFs participate in the tumor–immune cell crosstalk. Normal fibroblasts retard tumor growth, but some subsets of CAFs may accelerate cancer cell proliferation, invasion, enhance drug resistance and reduce anti-tumor immunity [97]. Most of the CAFs are thought to appear from normal fibroblasts due to abnormal activation by cancer cell stimuli [34,101,102]. A possible way for cancer cells to transform various stromal cells into CAFs is direct contact between the two cells [103], with subsequent transfer of either signal molecules or vesicles (see below).

CAFs most possibly participate in cancer invasion and metastasis by using reciprocal signaling between CAFs, cancer cells and the ECM through direct contacts, secretion of cytokines or EVs. For example, myofibroblastic CAFs (myCAFs) may directly interact with malignant cells; on the other hand, inflammatory CAFs can be more distant from the cancer cells and, possibly, are being reprogramed by the secretion of IL-6 and other cytokines [104]. However, in 2017, Öhlund et al. demonstrated that some of myCAFs mainly positioned close to tumor foci and possibly required juxtacrine interactions with cancer cells for their formation [105,106].

To better understand the pathways of information exchange between cells, it is important to evaluate the distances separating cells from each other in tumor compartments. To date, the distance between immune cells and stromal cells in these fields is not well understood [19]. On the other hand, the distance between CD8+ T cells and tumor cells and the distance between Tregs and tumor cells were estimated by Mezheyeuski and coauthors [107]. Generally, total T-cell infiltration was comparable between the CT and the IM: 349.2 cells/mm^2^ vs. 359.6 cells/mm^2^, respectively. CD8+ T cells in the CT were more clustered than in the IM (average distance between cells: 24.8 μm vs. 61.2 μm) and located closer to tumor cells (39.4 μm vs. 104.6 μm). Tregs were found to be located farther away from tumor cells in the CT than in the IM: (221.0 μm vs. 164.8 μm). In general, immune cell quantity was maximal at a distance of approximately 15–30 μm from tumor cells (typical eukaryotic cell size is from 10 to 30 μm) [19,107]. Also, FoxP3+ TILs were mostly located within a distance between 30 and 110 μm of CD8+ T cells [108,109]. Finally, in human breast carcinomas, the distribution of distances between carcinoma cells and nearest CAFs was on average 25 μm [110].

Summarizing this incomplete and scattered information, we can, however, conclude that both immune cells from each other and immune cells from cancer cells in tumor compartments are located at a distance of 30–100 μm. This distance is quite surmountable with the help of TNTs, but the question arises of how they, in an environment filled with an extracellular matrix, reach each other in order to form close contact, 2–20 nm, for the formation of gap junctions or synapses. This will be discussed in the following sections. Perhaps the exploratory systems of cells, for example filopodia, are involved in this process, and help cells come together at such a close distance. One can make a cautious assumption that rapprochement can occur after the formation of nanotubes due to their contraction, although it is usually believed that, on the contrary, tubes arise after the synapse (see, for example, [71]).

### 3.3. Fibroblasts, Immune and Cancer Cells Interactions

Epithelial cancer cells and components of the TME do not physically interact before BM degradation [42]. At this point, the cancer cells turn invasive, and physical communication between them and stromal cells participates in the metastatic process [38]. When it starts, cancer cells possibly expose on the surface adhesion molecules, such as cadherins, integrins, connexins and others [42], and their contact with neighboring cells and immune cells and CAFs form, possibly, synapse-like connections. Physical cell–cell communication between fibroblasts and other cell types can influence their chemical communication with immune cells [42,111]. There is only indirect evidence for the formation of synapse-like structures between CAFs and cancer cells or immune cells. The main one is associated with the expression of an inhibitory receptor expressed by T cells as a synapse component, Programmed Cell Death Protein 1 (PD-1), whose main ligand PD-L1 is expressed in cancer cells and surrounding stromal cells [112,113,114,115]. Until recently, PD-1 was considered only expressed on the surface of immune cells, whereas its ligands, PD-L1 and PD-L2, are only expressed in tumor cells. However, recent studies revealed the intrinsic expression of PD-1 in melanoma and many other cancers [113]. Therein, the close proximity between PD-1–PD-L1 and ISs is required for PD-L1 function to disturb the interaction between the TCR and major histocompatibility complex (MHC) [116].

Interestingly, the cis-interaction between PD-1 and PD-L1 was reported to occur if both were expressed on the same cell surface [117], which indicates their close positioning on the cell membrane [62]. T cells also interact with a number of cell surface ligands exposed by CAFs, including JAM2, OX40L and PD-L2, thus significantly increasing the duration of contact time between T cells and CAFs [118].

All these data clearly suggest that, at least where the PD1–PD-L1 interaction is detected, the formation of a synapse-like structure is very probable, including the formation of clusters of ligand–receptor pairs. Thus, during the interaction of CAFs with immune and tumor cells, in addition to TNTs, synapses are most likely formed that can serve as supramolecular targets of next-generation chemotherapeutics. Considering the co-expression of PD-1 and PD-L1 on many types of TME cells such as on tumor-infiltrating macrophages, myeloid-derived suppressor cells and dendritic cells, one can suggest that synapse-like interactions may be rather general in the tumor microenvironment. Note that many authors suggest the existence of other types of direct contact between TME cells and cancer cells, for example, gap or adherence junctions [61].

### 3.4. Communication of the Cancer and Microenvironmental Cells—A Supramolecular Target for Chemotherapy

An ongoing wheelspin of molecular targeting in clinical oncology [2] may be related to a crisis of the paradigm of the “magic bullet” theory [119]. The extreme cancer heterogeneity and the extreme complexity of the process of its molecular evolution, and the unimaginably complex metastatic finale, themselves create an intractable complexity. But this complexity is due not only to the participation of a huge number of processes and components, but also to the overwhelming unpredictability of the emergent properties of the system, which arise from their innumerable interactions and form fundamentally intractable problems [120].

Faced with this extreme complexity, the “magic bullet” concept does not appear to be fully adequate, and the old-fashioned chemotherapy still remains an unavoidable therapeutic option wherein surgery and/or radiation therapy cannot be effective despite its side effects on the patient’s health [16]. However, some new ways of using chemotherapy can be mentioned; for example, the use of epigenetic modifications as the chemotherapeutical target [121,122]. In addition, combination chemotherapy, which combines independently acting anticancer drugs, overcomes tumor heterogeneity to a certain extent [17,123]. In recent years, immunotherapy has become highly popular in the treatment of tumors. However, most patients do not respond to treatment or develop resistance. Thus, in order to achieve a better therapeutic effect, the combination of immune checkpoint inhibition and other therapies is one of the fruitful strategies [14]. However, an alternative universal and reliable concept of overcoming tumor resistance to treatments could be related to targeting the tumor microenvironment [15,119]. Analysis of the current studies in the field shows that the benefits of nanoparticle-based therapy were marginal compared to conventional chemotherapy [124].

At the same time, simultaneous nanoparticle-mediated delivery of temozolomide, and OTX015, an epigenetic inhibitor of bromodomain-containing protein 4 (BRD4), can block the tumor expression of PD-L1 and make glioblastoma cells a target for chemotherapy and immunotherapy [125].

In this connection, we will return to Hanahan’s paper, which we cited above [2]: “A military battlespace is a strategic approach that takes an integrative, holistic view of war, incorporating information about the enemy’s characteristics and armamentarium, precise topographical maps of all potential battlefields and war zones… The metaphorical war on cancer needs to adopt an analogous cancer battlespace plan, integrating knowledge about similar variable…, including: a census of a cancer’s variously specialized cells, the basis of their corruptions (e.g., genetic mutations, reprogrammed regulatory circuitry), and their lines of communication…”.

Analysis of such a strategic plan as applied to a tumor, combined with the principle of Occam’s razor, which recommends choosing the most economical path to solve a problem, and the analogy with military operations, suggests that the destruction of the lines of communication that turns a cancerous tumor into a kind of parasitic living organism may be a very effective way to destroy the tumor.

As we tried to demonstrate above, the arsenal of intercellular communications is diverse and penetrates all elements of the existence of the tumor “organism” [76,126]. The list of players implicated in cell–cell recognition and adhesion has grown to include the cadherin superfamily comprising classical, atypical- and proto-cadherins, nectins, connexins, lectins, eph/Ephrin and others. Such a rich palette of adhesion proteins has the potential to provide radically different effects upon cell–cell contact, from repulsion to adhesion and everything in between [127,128]. A total of 66% of cancer drugs listed in the DrugBank database target the surface proteins. A plasma membrane with its embedded proteins creates the environment for all these proteins to function cooperatively, thus achieving the optimal physiological output [129].

We will discuss synapses and synapse-like structures as attractive supramolecular targets. Although TNTs and the TM have been considered potential drug targets, we are still very far from translating these ideas into clinical practice [26,130,131].

As mentioned above, the development of T cell-mediated immunity includes the assembly of the ISs—the complicated interface between the T cell and the antigen-presenting cell [40,70,76,77,126,132,133,134,135,136,137,138,139,140]. The knowledge acquired to date about the mechanisms of IS assembly underscores this structure as a robust pharmacological target [126]. The above assumption that direct contact occurs not only between tumor and immune cells but also between tumor and other stromal cells [41] opens a new platform for a more universal chemotherapy.

## 4. Simple Principles of Specific Chemical Effects on Synapses


***Sola dosis facit venenum (“Only the dose makes the poison”)***
Theophrastus Philippus Aureolus Bombastus von Hohenheim (Paracelsus)

With all the differences in synapses, they retain some common properties. First, connections between cells are provided by the interaction of clustered protein ligands and receptors. Second, these connections are located close to each other. And finally, the underlying collective interaction is cooperative in nature and forms a common functional unit. In addition, synapses are also a limited space providing a focused cell–cell exchange of signaling molecules and particles, such as cytokines, growth factors and signaling vesicles. Consequently, synapse destruction may lead to multidimensional functional tumor collapses. Furthermore, the emergence of tumor resistance to this effect is very problematic. This effect could be universal for various tumors. The IS has already been indicated as a robust pharmacological target [70,126]. The different activities of molecules involved in IS assembly and function in the same local and narrow space provide the necessary platform for a supramolecular strike at a “single target” [70,126].

**Figure 3:**
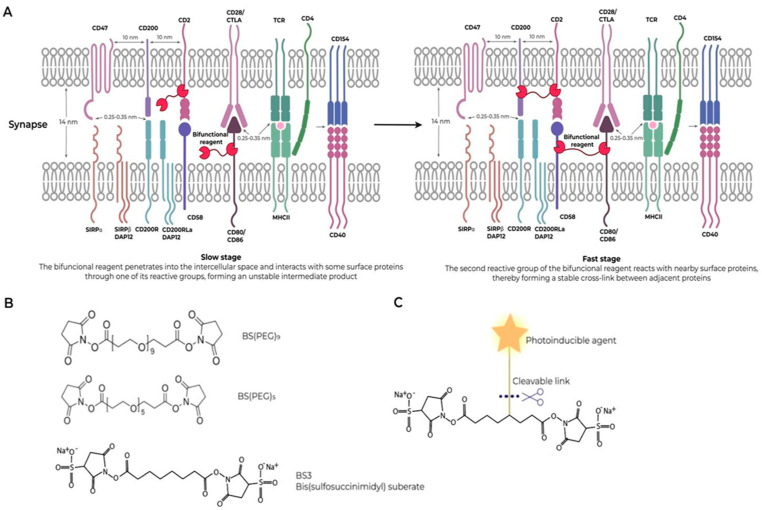
The rationale of cancer synapse-based chemotherapy. (**A**) A simple illustration of a two-stage reaction of a bifunctional crosslinking reagent with a protein cluster: the first stage, searching for a cluster, is slow; the second reaction inside a cluster with other protein neighbors is fast. (**B**) Examples of crosslinking agents. (**C**) A simple illustration of a bifunctional crosslinking reagent containing the (photo) activated group, shown as a star, and a cleavable link. The cleavable link can be used to deliver the active agent into the cell (see text).

The clustering of interacting components at the cell–cell interface at the synapse creates four unique opportunities to increase specificity while decreasing agent concentration:

1. Based on simple kinetic considerations, the probability of damage to at least one component of the cluster by a certain reagent specifically interacting with proteins exceeds the probability of damage to a single protein on the cell surface or in the extracellular matrix in proportion to the number of proteins involved in synapse formation. It becomes possible to drastically reduce the dose of the reagent hundreds of times and achieve its synapse-specific action, thus realizing Paracelsus’ concept that “substances poisonous in large doses can be curative in small doses” [141].
2. The specificity of the reagent can be further increased by using crosslinking reagents. A crosslinking reagent is a chemical compound that is used to covalently connect two functional groups of one or more molecules, particularly proteins, to each other (intra- or intermolecular). In this case, the reagent, having been bound to one of the components of the synaptic cluster, almost automatically reacts with one of the neighboring ones on the same surface or on opposite surfaces. This makes the modification durable (Figure 3A).
3. Non-cell-permeable crosslinkers can be used, which further increases specificity by limiting modification to only surface-exposed molecules. Examples of crosslinking agents are presented in Figure 3B.
4. Finally, one can enhance the effect by attaching an activated reagent to the crosslinker through a cleavable connection, which, after crosslinking, can be activated and separated from the crosslinker and, upon entering the cell, disrupt its vital functions (Figure 3C).

Figure 3 demonstrates one possible strategy for using the above features of synapses for cancer therapy.

To summarize, the proposed approach uses a chemical attack on the vulnerable communication field of the cancer with the environment by blocking the synapses formed by cancer cells and cells of the immune system and the synapse-like contacts of fibroblasts with cancer cells.

## 5. Instead of a Conclusion: Immunochemotherapy as a General Strategy Aimed at Poorly Fortified Areas of Cancers

It is now evident that the current gene-targeted cancer therapy paradigm must be evolved to a more effective one. To beat cancer, largely desperate attempts to influence the quickly transforming intracellular interactome of cancer cells could be replaced or complemented by the efforts focused on the interactions between cancer cells with the surrounding microenvironment. The survival of cancer cells depends on their interaction with their TME. Disruption of these connections, which are vital for the development and survival of tumors, is a difficult but promising and realizable task. A major example of the practical utility of this concept is tumor checkpoint immunotherapy. However, this approach destroys only a few of the multiple contacts of cancer cells with the TME, and theoretically this may be one of reasons why a significant fraction of patients does not respond to checkpoint immunotherapy. More tumor–TME interactions should be disrupted to allow for the development of more inclusive therapeutic strategies.

In some ways, an ideology comparable to that described herein has been used in combining immune checkpoint therapy with so-called “weaponized” antibodies and tumor-targeting antibodies combined with toxic chemicals. Recently, a great success of such a strategy was reported in treating bladder cancer. First, the monoclonal antibody pembrolizumab, which is a repressor of synapse-embedded ligand PD1 hindering immune T-cells, was blocked (this is immune checkpoint therapy, which releases T-cells for antitumor activity). Then, the treatment with a bifunctional agent, antibody–drug conjugate (ADC), enfortumab vedotin was undertaken. Enfortumab is an antibody against a member of a family of adhesion proteins, nectins (nectin-4), exposed on the tumor surface [142]. Nectins are clustered on the surface of some tumors and are involved in the formation of synapses with immune cells that widely express their binding partners [143,144]. The nectin-targeting antibodies were coupled through a cleavable linker with a toxic chemical (monomethyl auristatin E, MMAE) that interrupts cell division by disrupting replication spindle microtubules [145].

In this strategy, as well as in our suggestions, the first blow is struck at the synapse: the therapy according to [145] blocked repressive synapse component PD-1; in our suggestion, it is a general blow directed at synaptic protein clusters. In the second blow, the authors used a toxin, MMAE, attached with a cleavable link to separate antibodies. In an alternative approach that could be suggested, a toxin which can be the same MMAE or any other toxic intracellular agent is a part of the same agent that destroys the synapse. Theoretically, the latter approach looks considerably more simple, universal and affordable.

It should be noted here that the chemotherapeutic destruction of cancer cells using, for example, suicide gene therapy can also lead to the destruction of metastases, as noted in earlier reviews and research papers [146].

It has been mentioned above that, at least in some cases, chemotherapies can augment tumor immunity, for example, by inducing immunogenic cell death [10,11,12]. Most likely, a similar general anticancer immune response should be expected using the proposed strategy of disrupting the cancer synapses. There is every reason to believe that the chemotherapeutic constructs proposed herein can be delivered intratumorally. Recently, this delivery principle was reviewed in detail and it was shown that intratumoral injections of anticancer drugs may be a viable strategy to achieve higher local drug concentrations while reducing the risk of side effects (see, for example, [147,148]).

In this connection, we would like to return to the highly important problem of the practical application of cancer therapy in the context of a rapidly growing human population, which we have already touched upon in the introduction and which is addressed, in particular, by one of the leaders of modern oncology, Douglas Hanahan [2]. His concern is understandable, as many beautiful and even brilliant technologies based on the excellent achievements of modern molecular biology and genetics have turned out to be extremely expensive. As for the cost, personalized drugs are breaking all records. In 2017, the FDA approved two CAR-T drugs, namely Kymriah and Yescarta [149]. However, the drug treatments are extremely expensive. For instance, a one-time infusion of Kymriah or Yescarta costs USD 373,000 for adults with advanced lymphomas. In the case of children and young adults with acute lymphoblastic leukemia, Kymriah treatment costs USD 475,000. Moreover, apart from the drug cost, many patients encounter severe side effects that may require them to stay in a hospital intensive care unit for weeks. Consequently, the overall treatment expenses per patient can exceed USD 1 million, for one course of therapy [150]. Recently [151], the top five most expensive FDA-approved gene therapies were published, and the least expensive position there costs USD 850,000, whereas the price of the most expensive one is USD 5,800,000. With such a high upfront price, only a small fraction of patients can manage to pay for the medications and, therefore, will possibly benefit from the new developments. Also, such costs leave low- and middle-income countries out of personalized treatment (for a recent review, see [152]). Thus, developing alternative ways of conducting chemotherapy using different fundamental principles and more inclusive financial models is strongly required. We believe that the approach outlined in this review would serve as a basis for a new generation of desirable, inexpensive and universal clinical procedures.

## Data Availability

Data sharing is not applicable.

## Abbreviations

BM: basement membrane; CAFs, cancer-associated fibroblasts; CTLs, cytotoxic T lymphocytes; ECM, extracellular matrix; EVs, extracellular vesicles; EMT, epithelial–mesenchymal transition; IM, invasive margin; IS, immunological synapse; MHC, major histocompatibility complex; MMAE, monomethyl auristatin E; MMP, matrix metalloproteinase; MTs, tumor microtubes; PD-1, Programmed Cell Death Protein 1; TCR, T-cell receptor; TC, tumor core; TICs, tumor immune cells; TILs, tumor-infiltrating lymphocytes; TME, tumor microenvironment; TNTs, tunnel nanotubes; TS, tumor stroma; TNC, tenascin C.
